# Challenges Associated With the Use of Electronic Health Record Systems for Health Service Delivery in Ghana

**DOI:** 10.63116/XQPG1557

**Published:** 2026-02-12

**Authors:** Edward Agyemang, Kobina Esia-Donkoh, Addae Boateng Adu-Gyamfi, Emmanuel Kusi Achampong

**Affiliations:** 1 Ministry of Health, Ghana; 2 Department of Population and Health, Faculty of Social Sciences, College of Humanities and Legal Studies, University of Cape Coast, Ghana; 3 Department of Medical Education and IT, School of Medical Sciences, College of Health and Allied Sciences, University of Cape Coast, Ghana

**Keywords:** electronic health records, Lightwave Health Information Management System, software, hardware, challenges

## Abstract

**Background:**

Globally, digital health holds immense potential for transforming health-care delivery and improving patient outcomes, including enhanced care, increased mobility of records, reduced costs, and efficient recordkeeping. In this study, the authors sought to explore the challenges associated with the use of the electronic health record (EHR) Lightwave Health Information Management System (LHIMS) for health service delivery in Ghana.

**Methods:**

The authors used a nonprobability purposive sampling technique (expert sampling) to sample 30 unit heads from the study population.

**Results:**

The software challenges included inconsistency in report generation and a lack of standardization in system features, functions, and data capturing. The EHR (LHIMS) is also dependent on computer devices, and inadequate or malfunctioning devices pose a challenge to its effectiveness. Power interruptions can disrupt the work process and even affect patient outcomes.

**Conclusions:**

The challenges associated with the use of the EHR (LHIMS) in Ghana are issues that could have been resolved at the beginning of the implementation of the EHR if all the players had been involved in the process.

## Background

Globally, digital health holds immense potential for transforming health care delivery and improving patient outcomes, including enhanced care, increased mobility of records, reduced costs, and efficient recordkeeping.^1^ However, implementing electronic health records (EHRs) can be challenging, especially in Africa, where the continent grapples with various technological and technical challenges. Many African regions, Ghana being no exception, face inadequate technological infrastructure, including limited access to reliable electricity and internet connectivity. Without a stable network, accessing and updating patient data in real time, which is critical for an efficient EHR system, becomes challenging.

Financial resources pose a major barrier to EHR adoption worldwide. The initial investment and maintenance costs can be daunting for organizations, discouraging health care providers from adopting EHRs.^2^ This is particularly challenging for health care facilities in Africa with limited financial resources. The high setup cost in sub-Saharan Africa makes EHR implementation difficult for providers.^3,4^ Cost and funding also present significant challenges to EHR usability, hindering the effective use of these systems in patient care and management. Budget constraints further impede the adoption of alternative power solutions and essential resources, making it challenging to sustain digitally implemented EHR systems.^2^

Interoperability remains a challenge in EHR usage, with the lack of standardization and cultural barriers in the health care system impeding data exchange and utilization.^5^ Health care providers using different EHR systems find it difficult to achieve seamless interoperability. Inconsistent data formats and standards further obstruct information exchange between facilities, negatively impacting patient care and data sharing. In addition, some health care providers selectively share patient health records with other EHR systems to gain a competitive advantage and control patient information for financial benefit and market dominance.^6^

The transition from paper health records to EHRs presents a barrier owing to changes in workflow. Clinicians accustomed to using paper records for years face challenges in adapting to the new electronic system.^5^ Workers may require training to cope with this transition effectively. Implementing EHRs can lead to delays in completing tasks, as employees may have to wait for patient allocation or physician information submission, disrupting the facility’s workflow.^7^ The shift to EHRs can result in an increase in time spent on computers to check medical records compared with paper records.^8^

Clinicians’ level of technology literacy and skills can be a barrier to the adoption of EHRs in health care systems. A lack of knowledge in operating EHRs has been identified as a hindrance to their use.^9^ Technological literacy and skills, including typing proficiency, were found to affect EHR adoption.^10^ Interestingly, technologically inclined individuals were not always satisfied with EHR use. Poor literacy and technology skills, along with insufficient training, were reported as barriers affecting EHR usage.^11^

Certain health care facilities face challenges in resolving technical issues owing to the lack of skilled IT personnel, resulting in delays. This issue is particularly evident in Ghana, as evidenced by a study,^12^ which highlight difficulties with maintenance and support. Although these studies provided valuable insights into the challenges with EHRs in Ghana and other countries, they did not specifically address the challenges associated with the use of the Lightwave Health Information Management System (LHIMS) (an EHR), which the authors aim to investigate in the current study. In this study, the authors sought to explore the challenges associated with the use of the EHR (LHIMS) for health service delivery in Ghana.

## METHODS

Ethical approval for the study was granted by the Ghana Health Service Ethics Review Committee (GHS-ERC:011/07/21) and the Institutional Review Board of the Cape Coast Teaching Hospital (CCTHERC/EC/2021/095). Each participant received an informed consent form detailing the aims of the study and guaranteeing confidentiality and anonymity. The form further highlighted that participation was entirely voluntary and that respondents could discontinue their involvement at any stage without facing any repercussions.

### Study Design

The authors adopted a phenomenological research design to investigate the EHR phenomenon and to gain insights into health professionals’ lived experiences with the challenges associated with the use of the EHR in Ghana’s health care delivery system. The target group for this study was health professionals working in facilities that use the EHR for service provision. This group of professionals was purposively selected, as their consistent engagement with the EHR was expected to provide them with adequate familiarity and experiences to offer informed perspectives on its functionality and effectiveness.

### Sampling Technique

The authors used a stratified probability sampling approach to select the health facilities included in this study. This method helped to identify and select homogenous subpopulations with potential respondents who were similar in age, sex, education, work experience, and professional characteristics.

The authors used a nonprobability purposive sampling technique (expert sampling) to sample 30 unit heads from the study population, including 6 prescribers, 8 nurses/midwives, and 16 auxiliary staff. The unit heads were in a unique position to offer insights into the management and use of the EHR (LHIMS) at the facility. They were privy to specific details that might not have been accessible to other professionals and frequently served as representatives for their subordinates during consultative meetings. It was for this reason that purposive sampling was used in their selection as key informants for the study. At a point during the interview, new information or new insights were no longer being gained. It was at this moment that the interview process ended, as there was already enough information regarding the use of the EHR (LHIMS). This procedure was supported by Cresswell and Poth,^13^ who stated that, as previously noted by Shenton,^14^ a sample size of 5 to 30 people can be sufficient to reach saturation in qualitative research.

### Data Collection

An interview guide was developed to elicit data from the unit heads at the 10 health facilities using the LHIMS-EHR for their operations. We adapted questions from an interview guide developed by the Michigan Public Health Institute on EHR implementation.^15^ It probes health facility administrators, clinical staff, implementers, and office staff across a health care system. Some of the adapted items on the interview guide included users’ perceptions of EHRs. The interview guide contains questions on the general use of EHRs, privacy and security, interoperability, system complexity, high start-up cost, workflow changes, literacy and skill in technology, and reliability and stability of software, hardware, and computing network. The interview guide covered all these areas, including usability and financial challenges. Tablet computers were used for the interview recordings.

### Data Analysis

The Colaizzi-style^16^ approach of reading the descriptions, using existing relevant sentences, and creating interpretations was adapted. The approach entailed categorizing created concepts into topic clusters, exhaustively describing the researched phenomenon of LHIMS-EHR, and confirming the comprehensive description by each participant. According to Morrow et al,^17^ the Colaizzi style has significant potential for qualitative research, particularly for studies using the phenomenology design. To comprehend the participants’ experiences using the LHIMS for health service delivery, we first listened to the audiotapes and read the transcripts of the various interviews.

In the second step, we culled sentences and phrases that were pertinent to the challenges with the LHIMS-EHR and gathered extensive information about their lived experiences. To respond to the study questions and create an interpretation of the participants’ lived experiences with the use of the LHIMS-EHR, we deduced meanings from these noteworthy remarks and phrases in the third step. In addition, we gleaned themes and categories from the data and combined them to produce a thorough explanation of the challenges associated with the use of the LHIMS-EHR. When regularities and patterns started to show in the data, we continued to analyze the data until we concluded that the saturation threshold had been achieved. Finally, by letting themes develop from the data, pondering about the data, and identifying recurring themes from certain quotations, we were able to gather and maintain truly subjective data.

## RESULTS

In total, 30 participants were recruited for the study; participant characteristics are presented in Table 1. Eighteen participants were 30 to 39 years old, 17 participants were male, 19 participants were married at the time of the interview, and 22 participants had working experience of <10 years. Sixteen participants fell under the auxiliary category (such as a pharmacist, laboratory technician, or medical record technician), 8 participants were identified as nurses/midwives, and 6 participants were prescribers (medical doctors and physician assistants).

**Table 1.** Sociodemographic Characteristics of Participants

**Category**	**Participants (n = 30)**	**Percentage (%)**
Sex		
Male	17	56.7
Female	13	43.3
Age		
30–39	18	60
Above 40	12	40
Marital status		
Single	11	36.7
Married	19	63.3
Work experience		
<10 years	22	73.3
>10 years	8	26.7
Profession		
Auxiliary	16	53.3
Nurses/Midwives	8	26.7
Prescribers	6	20

### Technical Issues

The interviews revealed significant technical challenges that health care professionals encounter using the EHR (LHIMS). Among these challenges, unforeseen equipment problems, such as hardware failures and software bugs, emerged as one of the major hurdles, impeding the smooth execution of desired actions. Software and hardware challenges were also identified. These challenges elucidate the complexities faced in effectively integrating the EHR (LHIMS) and underscore the need for comprehensive solutions to ensure successful implementation and enhance health care practices.

#### Software challenges

Under the category of software challenges, the foremost issue identified was the inconsistency in report generation linked to the utilization of the EHR (LHIMS). During the interviews, participants, particularly health information officers, reported that the EHR (LHIMS) produced varying results for the same report. Specifically, when running a report on the number of clients attended to at the outpatient department (OPD), the system generated inconsistent outcomes. Several participants expressed their concerns regarding this matter, stating the following:

I have reservations about the system’s accuracy as some of the data generated after entry appears to contain errors. Specifically, the reported cases of certain diseases, such as malaria, seem to represent only about 1% of the actual occurrences. Due to these discrepancies, I find myself manually verifying the data at the end of each month, cross-referencing it with the system’s provided information to ensure accuracy [A staff at the health information unit of one of the facilities].

We have observed disparities in certain reports concerning morbidity at the OPD. The information provided is not entirely accurate. We generate the correct data by cross-referencing with paper records whenever such discrepancies arise. This approach enables us to obtain accurate and comprehensive reports for every disease [A staff at the health information unit of one of the facilities].

There is always a problem of inconsistency in the kind of report generated by the LHIMS. You can generate a report right now on a particular condition and when you run another report on the same condition the next minute, you will get a different result [A staff at the OPD of one of the facilities].

The second software issue centered on the standardization of system features and their utilization. Despite the interoperable nature of the EHR (LHIMS), a deficiency in standardization was evident in system features, functions, and utilization. Notably, specific facilities had tailored their EHR (LHIMS) software to align more with their distinct requirements. However, the implementation of these customizations, unless executed meticulously in adherence to clear guidelines and standards, carries the risk of undermining the system’s integrity and data capturing.

When LHIMS was initially introduced, it featured 3 parameters. However, the system is designed to be adaptable, allowing users to customize inputs according to their preferences. Upon realizing that LHIMS did not provide the full range of parameters we required, we promptly informed the IT managers. They responded by redesigning the system to meet our specific needs. Flexibility is a key aspect, and by communicating with IT, they can adjust it exactly as per our preferences [A staff at the laboratory unit of one of the facilities].

It appears that the LHIMS in this facility differs from the one I used at my previous workplace in several ways. There are certain features here but not at my previous health facility and vice versa [A nurse at one of the facilities].

The design of LHIMS in this facility is not the same as in other facilities. They have customized it and picked certain things out. It has been designed to suit the laboratory in this facility [A staff at the laboratory unit of one of the facilities].

During the interview, another challenge that arose was facilities referring patients through the paper-based approach. Participants indicated that they write referral notes and physically hand them over to the referred clients. According to the participants, they were unaware of such processes in the EHR (LHIMS). Some of them shared their experiences, narrating the difficulties and inefficiencies they encountered owing to this issue.

Patients merely provide a referral note, and I am not aware of any option or feature on LHIMS that would allow me to initiate a patient referral to another facility with a simple click. Upon enquiry, that functionality does not exist even though patients from our facility use the same LHIMS number [patient number] in other facilities on the LHIMS [An optometrist in one of the facilities].

Another respondent also said this:

Regarding patient referral, we manually include the pertinent details that the prescribers should be aware of on paper. The reason is that LHIMS lacks a specific feature to facilitate referrals. Consequently, we record the information on paper, which is then carried by the patient to the other facility [A staff member at the health information unit in one of the facilities].

The third issue under the software challenge revolves around the limited system features of the EHR (LHIMS). For instance, the participants indicated that the system does not capture some professional types, certain diagnoses, and some drugs. Respondents expressed concerns over the restricted list of diagnoses available in the EHR (LHIMS) and the use of unfamiliar names for certain conditions, making it difficult to locate specific health issues within the system. Consequently, they find themselves compelled to choose “near-diagnoses” or similar disease conditions. Below are some narrations provided by the participants regarding this matter:

At times, particularly when inputting a diagnosis, it can be challenging to find the exact option you want within LHIMS. As a result, you may have to select the next best or similar diagnosis from the dropdown options because the precise diagnosis you seek might not be available in the system’s dropdown menu [A physiotherapist in one of the facilities].

I am compelled to select near diagnosis. I find myself forced to choose a diagnosis that is close but not exact. For instance, when a patient visits, LHIMS is meant to assist in selecting the diagnosis and prescribing appropriate medication. However, that doesn't always happen smoothly. Sometimes, when searching for a specific diagnosis, it is not available in the drop-down menu. In such cases, I must opt for a closely related health condition for the patient; otherwise, the patient cannot access the pharmacy for the prescribed medicine [An optometrist in one of the facilities].

There were other issues reported by participants concerning user accounts of some professions on the EHR (LHIMS). This challenge was commonly reported among physiotherapists. One of them had this to say:

Access to the LHIMS is granted based on your profession, but unfortunately, our profession of physiotherapy is not included in the system. As a result, we are forced to enter as medical officers, even though that does not accurately represent our roles. This limitation significantly impedes our work efficiency [A physiotherapist in one of the facilities].

Similarly, laboratory technicians and radiographers from various health facilities expressed that the EHR (LHIMS) lacks comprehensive coverage of all the parameters required for a full blood count. Moreover, they are unable to upload X-ray results onto the EHR (LHIMS), resulting in limited data entry capabilities. Two of them shared their experiences as follows:

The LHIMS lacks the capability to capture all laboratory parameters effectively. For example, when conducting a full blood count, numerous essential parameters such as white blood cells (WBC), and hemoglobin (HB) are involved, yet the system does not offer a field to capture all of this data comprehensively. Instead, only 3 parameters can be inputted. These are WBC, HB, and platelets. Consequently, relying solely on these limited parameters for diagnosis might prove insufficient. In cases like antenatal care, where bleeding issues are a concern, other crucial parameters beyond those captured in the LHIMS must be considered by clinicians [A staff at a laboratory unit of one of the facilities].

The LHIMS at our facility lacks the functionality to upload scanned images or films from X-rays directly. While some other facilities have successfully integrated their analyzers and the LHIMS software, our facility does not currently have such a setup in place [Radiographer from one of the facilities].

Participants also expressed that the EHR (LHIMS) could not edit inputs after saving, and this challenge was reported across nearly all the health facilities included in the study. In situations where editing was not possible, they resorted to using notes as a means of communication with other EHR (LHIMS) users. These notes were attached to previous inputs, serving as additional information for anyone accessing a particular client’s records. However, participants highlighted that this practice posed a challenge, as not everyone might have the time or capacity to read the attached notes, especially when dealing with a large number of clients. Below are some narrations provided by participants to substantiate their claims:

In case of an error, my only option is to write a note to inform other system users who may require the information that the initial note contains inaccuracies. I can attach the corrected version to the note, but I am unable to edit or delete the existing note [A staff at OPD of one of the facilities].

In the LHIMS, you have the option to enter a counter note, but you cannot directly edit any previously entered mistakes. If an error is made, the process involves rewriting the correct information as an additional piece of data, rather than directly modifying the original input [A surgeon in one of the facilities].

Another limited system feature-related challenge is the inability of the EHR (LHIMS) to autosave information. Users of the EHR (LHIMS) complained that they always had to start from scratch anytime there was a power interruption, which led to the loss of information. They shared their views that the EHR (LHIMS) should have the autosave function to ease their task. The following were their exact expressions:

In this area, power cuts are frequent, and unfortunately, we do not have a UPS to mitigate their impact. During a blackout, any unsaved information gets lost, forcing us to painstakingly re-input or retype all the data. This repetitive process can be extremely exasperating and time-consuming, often causing frustration and annoyance [A physiotherapist in one of the facilities].

In addition to the delay caused by writing notes in the system, there’s the added frustration of losing significant progress during power outages. Imagine typing a whole page of content, only for everything to vanish due to a sudden power cut. It would be beneficial to have an autosave function in place, ensuring that even if the power unexpectedly goes off, the system can retain the entered information and prevent such losses [A midwife in one of the facilities].

In addition to the EHR (LHIMS) lacking an autosave feature and the ability to edit inputs, discussions with participants highlighted another issue—the absence of a previewing option before saving client information. This limitation has been a significant challenge for them. Participants expressed the need for a previewing option that would enable them to review and read through the entered data before finalizing the save. Such a feature is crucial in preventing input errors. Some of the participants shared their experiences, saying:

The system does not permit you to review and confirm the accuracy of the information you have entered before saving it [A midwife in one of the facilities].

The option to preview our entries before saving is completely absent. The system must get updated to incorporate this feature, as it would be immensely beneficial in preventing minor mistakes and errors [A doctor in one of the facilities].

#### Hardware challenges

The second major theme that emerged as a challenge under technological issues was related to computer hardware issues. This encompassed a range of related problems, including insufficient and malfunctioning computer devices. The flawless functioning, contentment, efficiency, and effectiveness of the EHR (LHIMS) hinged on the performance of these computer devices. Their availability and optimal condition were pivotal in ensuring smooth operations and accurate data capture. During the discussions with the participants, it became evident that the inadequacy of desktop computers and tablets posed considerable challenges in using the EHR (LHIMS) effectively. For instance, numerous health professionals expressed their frustration with the need to queue and manually enter patient details into the LHIMS owing to the lack of sufficient computers.

When you come to my unit, there is only 1 computer available. However, we are about 6 individuals in this unit, and it is challenging for all of us to share a single device. This situation is not appropriate and hampers our efficiency. The need to queue and take turns for data entry significantly delays our service delivery. To be frank, some people even neglect to enter patients’ details into the LHIMS due to these difficulties [A midwife in one of the facilities].

The computers we currently have in this facility are insufficient. LHIMS requires a computer to operate, and unfortunately, we do not have enough computers, making it challenging to use the system effectively. It is unrealistic to expect about 10 people in a unit to share 1 computer; such an arrangement is simply not feasible. We need additional computers to fulfill our work requirements properly [A nurse at OPD of one of the facilities].

Furthermore, some participants shared a similar viewpoint regarding the challenges they faced owing to computer breakdowns and malfunctioning peripheral devices, such as mouse and keyboards. They expressed that these issues hindered their ability to use the system as intended. According to their feedback, when these devices are not promptly fixed, it creates difficulties in using the EHR (LHIMS) effectively, leading to potential lapses in capturing some patients’ records on the system.

We have approximately 4 computers, and unfortunately, none of them are functioning. Since LHIMS requires a computer to operate, the nonfunctionality of these devices makes it challenging to use the system effectively [A midwife in one of the facilities].

The computer keyboard is not functioning properly, and the mouse is also causing issues. A computer without a functional mouse cannot be used effectively. It is challenging to carry out tasks without a proper keyboard and mouse. These are the challenges we face in using LHIMS [A nurse manager in one of the facilities].

The delay in repairing broken-down devices emerged as a common challenge identified by participants across various health facilities. They expressed their concern that the extended period it takes to fix these devices forces them to resort to using the paper system. This, in turn, creates an additional burden for users of the system, as they have to later transfer the data onto the EHR (LHIMS), leading to a double task. Consequently, this perceived challenge was highlighted by several participants. Below are some of the narrations provided:

You see this computer [shows researcher the device] has been inactive for about 3 months now. When you report it to the administration, they only promise to come and fix it, but nothing happens afterwards. How are we supposed to use LHIMS on a broken device? Besides, the devices are already inadequate, and the broken ones are not fixed promptly either [A nurse in one of the facilities].

Our computers are not fixed when they develop faults. Many devices are broken down, and there are no attempts to repair them. Even when they eventually get fixed, it takes an extended period. All these factors pose challenges to the effective use of LHIMS [A dispensary assistant in one of the facilities].

#### Power interruption

A stable and continuous availability of electricity significantly contributes to the efficient functioning of the EHR (LHIMS). Through discussions with various health professionals, it became evident that power outages pose a major challenge in using the EHR (LHIMS). Participants expressed their concerns that whenever there is a power outage, service delivery comes to a halt, and patients may experience delays until an alternative power supply is provided. This interruption occurs because they rely on the EHR (LHIMS) to capture patient records. The reality is that some facilities lack a standby generator or power plant. Consequently, when the power goes out, they are forced to pause their operations, as their desktop computers rely on electricity to function. Even facilities equipped with generators may encounter issues such as fuel shortages or delays in turning on the generator by those responsible. Some participants shared their experiences as follows:

My facility has a generator, but unfortunately, we often have to wait for a while until someone goes and turns it on for us. The security personnel are responsible for activating it, but sometimes they fail to do so promptly [A midwife in one of the facilities]

During power outages, everything comes to a halt. We are left waiting until the power is restored because, without it, we cannot enter patient records into the system. We have no choice but to wait or resort to using paper records, which we later have to input into the system once power is restored. This situation creates double work for us, and it can be quite frustrating [OPD in-charge in one of the facilities].

In addition, some participants voiced their concerns about their facilities lacking an uninterrupted power supply (UPS) to sustain or serve as backup power during instances of inadequate voltage or power outages. Two of them shared their experiences as follows:

When the power goes off because we do not have a UPS, if you have not saved your information, you will lose it. As a result, you need to start all over again. Additionally, if there are issues with the generator, the patient might have to wait for about an hour until it starts working again. These are just a few of the problems associated with LHIMS [A physiotherapist in one of the facilities].

When the power goes off, having a UPS to retain power is crucial. However, currently, there is no UPS available. Most of the devices are not running on UPS, and as a result, when a power outage occurs, it has a significant impact on the system. This situation poses a major challenge for us [A pharmacist in one of the facilities].

#### Unstable local area network

Participants also highlighted internet interruptions as another technology-related challenge. They explained that the EHR (LHIMS) relies on internet accessibility and utilization, and any disruption in the service makes the delivery of health services challenging. In certain facilities, health professionals mentioned that even though internet service was available, it became difficult to access during the daytime. Some facilities were only able to use the internet at night. These were the accounts shared by some of the participants.

There are times when the internet works perfectly throughout the night, but there is no access during the day. Just imagine attending to numerous clients during the day, and the internet only becomes available at night. Those who came during the day would not be captured in the system, creating a significant gap in patient records [A midwife in one of the facilities].

Some health professionals reported that a slow network posed challenges to the effective and efficient use of the EHR (LHIMS). Facilities with a high number of patients revealed that slow network speed resulted in delays in service delivery, leading to long queues. Certain participants mentioned resorting to the paper system and later entering clients’ information into the EHR (LHIMS). Moreover, some also noted that internet speed slows down during the day, especially when it rains. Some participants expressed their experiences as follows:

Most of the time, the network becomes slow, and there are instances when you have to move around to find a stable network connection. I have personally experienced this situation before. There was a time when I had to leave my office and walk around until I found a better network speed to access LHIMS and serve medication to patients [A nurse at the eye clinic in one of the facilities].

The network is much faster at night compared to daytime. Throughout the whole day, the speed of the network can be very frustrating; it is notably slow. My colleagues have mentioned that it works faster at night. However, the question arises: How many people come to the hospital at night? Only a few people do [A midwife in one of the facilities].

#### Distortion of information after updates

Updates can prevent security issues and improve compatibility and program features. Software updates are necessary to keep computers, mobile devices, and tablets running smoothly, and they may lower security vulnerabilities. However, discussions with participants indicated that system updates sometimes lead to loss of information from the EHR (LHIMS) or mess up patients’ records.

There are instances when system updates lead to the distortion of information in the system. Upon checking, you may find that certain stored information is no longer accessible due to the update that was performed. In response to these issues, the Lightwave technical team has decided to avoid updating the system unless there is a specific problem that needs to be addressed [A pharmacist in one of the facilities].

There are times when updates are implemented, and therefore, the information on the system gets messed up. For example, you may see drugs physically present on the counter, but when you check the system, it incorrectly indicates that there are no drugs available [A pharmacist in one of the facilities].

### Human Factors

Using a specific system requires users to possess certain skills and expertise. From the analysis, an important theme emerged, indicating that service providers lacked the necessary skills and expertise, which acted as a barrier to the effective utilization of the EHR (LHIMS). Two major challenges were identified: inadequate training received on the EHR (LHIMS) and computer illiteracy among service providers.

#### Inadequate training on the EHR (LHIMS)

Inadequate skills on the part of users can pose significant challenges. During discussions with participants, it became apparent that many of them either received no training on the EHR (LHIMS) or the training they received was insufficient. Most participants reported that they were trained for only a day, which they considered inadequate. Some of them expressed their experiences as follows:

We do have training, but it does not happen frequently. Moreover, the training provided is a general session for everyone, and it only lasts for a single day. However, one of the challenges we face is that they do not remind us about updates [A staff member at the medical record unit of one of the facilities].

They only came to introduce the LHIMS to us and showed us how to enter data into it. Unfortunately, we did not receive any significant training on how to use it effectively [An optometrist at one of the facilities].

#### Computer illiteracy

Participants not only attributed the challenges they faced in using the EHR (LHIMS) to inadequate training but also recognized computer illiteracy as a barrier to its utilization. Despite receiving training or coaching from friends on how to use the system, older users found the system difficult to navigate because they lacked prior knowledge and skills in manipulating computers. Some of them expressed their experiences as follows:

Regarding age, I believe so because those who were born before the computer era may face certain challenges when using LHIMS. Perhaps, they lack the necessary computer skills that would enable them to use LHIMS effectively [An optometrist in one of the facilities].

All the workers in our facility, except for an elderly woman, can use LHIMS. She finds it difficult to navigate the system, most likely due to her age and lack of computer knowledge and skills [A midwife at Antenatal Care in one of the facilities].

The use of LHIMS is affected because not all the prescribers are proficient in IT. As a result, the elderly prescribers attend to the clients but rely on younger colleagues or nurses to handle the data entries for them, since they lack computer manipulation skills [A health information officer in one of the facilities].

## DISCUSSION

The implementation of EHR systems, such as LHIMS, has proven beneficial in enhancing interoperability, streamlining workflows, and improving access to patient data. This study adds to the growing body of knowledge by providing evidence from Ghana, highlighting how local infrastructural, software, and human resource constraints shape the success of EHR use. Recent work by Mensah et al^18^ and Acheampong et al^19^ corroborated these findings, demonstrating that although EHRs enhance data accessibility, their impact depends heavily on contextual enablers, such as reliable connectivity and system design quality.

The study demonstrates that interoperability within facilities can be achieved, as shown by the successful integration of consulting rooms, pharmacies, and X-ray units. This finding aligns with other research works showing that interoperability enhances care coordination, reduces duplicate tests, and strengthens communication between providers.^20,21^ However, the co-existence of parallel systems such as the Basic Laboratory and Information System alongside LHIMS undermines interoperability. This reflects a broader problem in EHR deployment, where a lack of system uniformity creates inefficiencies and risks for patients. By documenting these issues in Ghana, this study contributes practical insights into the importance of policy-driven standardization to support integrated digital health systems. This issue was emphasized by Donkor^22^ in the context of the Ghana Digital Health Strategy (2023–2027) and by Mugauri et al^23^ across sub-Saharan Africa.

The study highlights that limited system functions and outdated coding frameworks (eg ICD-10 instead of ICD-11) restrict clinical precision and force health professionals to make approximations. This mirrors findings from studies by Ajayi et al^24^ and Mukono and Tokosi^25^ that limited system design hinders adoption, and it is consistent with the work by Antor et al,^26^ who found similar usability and coding constraints at Komfo Anokye Teaching Hospital. Ranney^27^ further argued that the lack of standardized data architectures and metadata alignment across systems remains one of Africa’s most persistent EHR integration barriers. This research contributes to current practices by advocating for EHR design improvements tailored to clinical realities in low-resource settings, including expanding diagnostic libraries and enabling editing, autosave, and referral features.

Infrastructural gaps such as insufficient computers, unreliable power supply, and unstable local networks were major barriers. These challenges resonate with recent studies on EHR downtimes, which emphasize risks to patient safety and workflow disruptions.^5,19,20,23,28–30^ This study provides local evidence for investment priorities: procurement of adequate devices, backup power, and stable local area networks should not be considered optional but essential components of EHR rollouts. Similarly, Aggrey et al^31^ demonstrated that achieving functional interoperability at the University of Ghana Medical Centre required significant infrastructural and technical upgrades to stabilize networks and ensure data consistency across subsystems.

Human factors such as inadequate training and computer illiteracy remain central obstacles. Most users received only 1 day of training, and older staff often struggled with basic computer tasks, leading to reliance on paper-based workarounds. Recent studies confirm that continuous, role-specific training significantly improves user satisfaction and reduces errors.^18,32–34^ This study contributes to practice by emphasizing that training must be iterative, contextualized, and inclusive, particularly where computer literacy gaps are significant. Moreover, Ranney^27^ underscored that human resource development is a determinant of sustainable interoperability, suggesting that digital literacy should be embedded into health workforce curricula.

Collectively, these findings contribute to global EHR practice by showing that although the technical principles of interoperability, usability, and reliability are universal, their implementation must account for local realities of infrastructure, workforce skills, and policy alignment. Mugauri et al^23^ and Ddamba et al^30^ reinforced that contextual adaptation rather than direct adoption of Western EHR frameworks yielded more sustainable outcomes in African settings. Ghana’s experience thus provides valuable insights into how digital health transformation must balance technological ambition with sociotechnical readiness.

## CONCLUSIONS

This study highlights the multifaceted challenges of using LHIMS for health care delivery in Ghana, ranging from software limitations and lack of system standardization to hardware inadequacies, power instability, unreliable networks, and insufficient user training. These challenges are not unique to Ghana but resonate with barriers reported globally, reinforcing the notion that digital health transformations require holistic strategies that integrate technology, infrastructure, policy, and human capacity.

By situating Ghana’s experience within the global discourse on EHRs, this study provides context-sensitive evidence that can guide future implementation strategies, not only in Ghana but also in other low- and middle-income countries facing similar challenges. In doing so, it contributes to improving current practices by showing that successful EHR implementation requires not only the deployment of technology but also systemic investments in people, processes, and infrastructure.

## AUTHOR CONTRIBUTIONS

E.A., K.E., A.B.A., and E.K.A. were responsible for the conception and design of the study. E.A. was responsible for the design of the survey questions, the acquisition, and interpretation of data. E.A. conducted statistical analyses, drafted tables, and figures. E.K.A. wrote the original manuscript. All authors reviewed and edited the manuscript. The final version of the manuscript was approved by all contributing authors.

## DISCLOSURES

The authors have nothing to disclose.

## FUNDING

The authors received no funding for this research.
